# Rural digitalization and the multidimensional wellbeing of older adults in China: evidence from the active aging framework

**DOI:** 10.3389/fpubh.2026.1900178

**Published:** 2026-10-19

**Authors:** Hubang Wang, Qiaohan Liu, Yujie Liu, Shimin Wei, Meihan Zhu, Boyao Li, Hongli Wang

**Affiliations:** School of Statistics and Data Science, Jilin University of Finance and Economics, Changchun, Jilin, China

**Keywords:** active aging, double machine learning, level of digital development, synergy, wellbeing of rural older residents

## Abstract

**Background:**

Building a more inclusive digital environment is a key strategy for promoting rural digitalization, narrowing the digital divide, and enhancing the wellbeing of older adults in rural areas. However, despite its increasing relevance, research on how rural digitalization affects the wellbeing of older adults in developing countries remains scarce.

**Objective:**

This study aims to examine the relationship between rural digitalization and the wellbeing of older residents in rural areas, with a particular focus on its underlying mechanisms and heterogeneous effects.

**Method:**

Grounded in the World Health Organization (WHO) framework for active aging and the multidimensional measurement approach of the Active Aging Index (AAI), this study develops a comprehensive index for evaluating the wellbeing of older adults in rural China while accounting for the specific socioeconomic and contextual characteristics of rural areas. Using matched data from the China Provincial Digital Development Index (2016–2022) and the China Family Panel Studies (CFPS), to systematically analyze the relationship between rural digitalization and the wellbeing of the older adults in rural areas, as well as the mechanisms underlying this relationship, and the underlying mechanisms are explored through a double machine learning approach.

**Conclusions and discussion:**

Analysis indicates that rural digital development significantly enhances self-efficacy, expands social networks, and strengthens social trust among rural older residents. These factors constitute key mechanisms through which digital development is associated with improved wellbeing among rural older residents. The heterogeneity analysis further reveals that the positive impact of digitalization on wellbeing are more pronounced among older adults with lower educational attainment, those with moderate or low income levels, and those living with their children, and the positive correlation is stronger in China’s central and western regions. Overall, this study extends active aging theory to rural digital contexts in developing countries, thereby improving its contextual applicability, while also enriching the existing framework for evaluating the wellbeing of rural older adults and providing policy insights for promoting an inclusive digital society and a more equitable distribution of digital dividends.

## 1 Introduction

The wellbeing of older adults in rural areas is not only central to improving individual quality of life but also plays a significant role in strengthening rural social resilience and advancing the United Nations Sustainable Development Goals (1). Against the backdrop of rapid industrialization and urbanization, many developing countries are experiencing large-scale migration of the working-age population to urban centers, leading to pronounced rural “hollowing-out” and accelerated population aging. Rural communities face increasing challenges in ensuring adequate healthcare, promoting social participation, and providing equitable access to basic public services for older adults (2). The theory of active aging, advocated by the World Health Organization, moves beyond the narrow focus of traditional gerontology on survival and healthcare security. It is built on three core pillars, namely, health, participation, and security, and emphasizes that older adults are active agents who can contribute meaningfully to society and be integrated into social development processes (3). Because rural digitalization directly shapes older adults’ access to health management, social participation, and protection from online risks—corresponding precisely to the three pillars—this theory offers an ideal lens for analyzing how digitalization influences their wellbeing. In the meanwhile, this perspective offers a valuable analytical framework for reinterpreting the concept of wellbeing among older rural adults and identifying pathways to enhance it.

Currently, the global digital revolution is reshaping economic structures, public services, and patterns of social interaction while also offering new governance approaches and development opportunities to address the challenges of rural aging in developing countries (4). Compared with the relatively advanced level of digital application in urban areas, rural digitalization is more multifaceted, encompassing infrastructure development, grassroots governance strengthening, and universal public service provision. Therefore, its effectiveness is closely associated with improvements in the quality of life and overall wellbeing of older adults in rural areas (5). Existing research has primarily focused on analyzing digital technology usage behaviors, such as internet use and the adoption of smart devices (6). Recent research on digitalization, the digital divide, and the health and wellbeing of older adults in China has expanded significantly. Relevant studies have examined age-related digital divides and their socioeconomic disparities (7); urban–rural, gender, and health disparities in community-based digital divides among older adults (8); the interplay between the digital divide, digital support, and health-related quality of life among rural older adults (9); and the relationship between rural digitalization and health outcomes in this population (10). These studies provide a crucial foundation for understanding digital inclusion and its implications for the health and wellbeing of China’s older populations.

More importantly, existing studies predominantly focus on specific outcome variables, such as physical health, mental health, or health-related quality of life, with comparatively limited attention to core dimensions of active aging, such as social participation, social connectedness, and personal value realization (11). Furthermore, while previous research has largely examined the digital inclusion of older adults at the individual level, such as internet access, digital technology use, and the digital divide, this study focuses on rural digital development at the provincial level. Consequently, there are distinct differences in the measurement levels and conceptual scope of the two. Therefore, further research is needed to examine the relationship between rural digitalization and the multidimensional wellbeing of the rural older within the framework of active aging. As a developing country with one of the largest aging populations globally, China is characterized by a substantial older population, a pronounced urban–rural divide, and significant regional disparities. Against the backdrop of ongoing industrialization and urbanization, rural areas in China have long been confronted with the dual challenges of outmigration among young and middle-aged workers and an increasingly aging population. This distinctive context, where rapid digitalization coexists with deepening demographic aging, makes rural China a critical case for examining how rural digitalization influences the wellbeing of older adults.

This study adopts the WHO Framework on Active Aging as its analytical framework and focuses on two core objectives. First, the association between rural digitalization and the wellbeing of older rural residents is systematically identified. Second, it provides an in-depth exploration of the intrinsic mechanisms linking digitalization to older adults’ wellbeing, while also clarifying the heterogeneous differences in digital empowerment associations across different individual characteristics and regional development conditions. Addressing these issues will help identify the complex mechanisms linking rural digitalization to the wellbeing of older adults and expand the applicability of active aging theory within the context of rural digitalization. In terms of research design, this study distinguishes between the theoretical and measurement frameworks: the WHO Active Aging Framework provides the theoretical foundation for defining and analyzing the wellbeing of rural older adults, while the AAI serves as a reference for selecting and constructing the corresponding wellbeing indicators. Based on this, this paper draws on the multidimensional measurement approach of AAI and constructs a comprehensive wellbeing evaluation index system tailored to rural older adults using microdata from the China Family Panel Study. Methodologically, a double machine learning model is employed to estimate the association between rural digital development and the wellbeing of older adults, thereby providing a more robust examination of the mechanisms linking rural digitalization to the wellbeing of rural older adults.

Compared with existing studies, this study makes three key contributions. First, it extends the application of active aging theory within the context of rural digitalization by incorporating the wellbeing of rural older adults into a comprehensive analytical framework that spans health security, social participation, and developmental capacity. In doing so, it strengthens the theoretical foundation of research on the wellbeing of the older in the digital era and offers a useful framework for the localized application of active aging theory in rural settings across developing countries. Second, this study examines the mechanisms through which rural digitization is associated with the wellbeing of older adults in rural areas across three dimensions: self-efficacy, social networks, and social trust. It also conducts a heterogeneity analysis based on individual characteristics and regional development disparities, thereby providing a comprehensive understanding of the underlying mechanisms and differentiated impacts of digital empowerment. This offers a benchmark of comparable evidence for developing countries in formulating targeted and inclusive digital aging policies. Third, the study leverages double machine learning models to account for nonlinearity and high-dimensional confounders, thereby providing more robust causal evidence on the effects of rural digitalization on older adults’ wellbeing. By improving the reliability of these estimates, this approach helps clarify the mechanisms through which digitalization influences health, social participation, and personal value realization, offering both empirical and methodological contributions for research on digitalization and aging.

## 2 Literature review

### 2.1 The evolution of measures of wellbeing of older adults and the gap in rural digitalization

As a fundamental concept, the meaning of “welfare” continues to evolve alongside the expansion of research perspectives. Existing studies generally agree that wellbeing is reflected not only in individuals’ subjective evaluations of their lives but also in their objective living conditions and capacity for development (12, 13). While the traditional notion of “living conditions” primarily emphasizes objective factors such as income, health status, and material resources, the concept of wellbeing places greater emphasis on an individual’s overall quality of life within their social context, the realization of their potential, and their perceived sense of value. This underscores the integrated relationship between objective living conditions and subjective experience, providing an important theoretical foundation for the shift in research on older adults from a focus on “subsistence security” to “quality of life.”

The measurement of wellbeing of older adults as a specific extension of the concept of wellbeing among older adults draws on multidisciplinary perspectives, including health economics and social gerontology, and incorporates multiple dimensions such as health status, psychological functioning, self-perception, and life satisfaction. However, traditional aging research has tended to emphasize objective living conditions such as income security, healthcare provision, and physical health, prioritizing the fulfillment of older adults’ basic survival needs while paying less attention to higher-order needs such as social participation, personal value, and developmental capacity (11). Although traditional scales, such as the SF-36, capture both physical and psychological dimensions, their applicability in rural contexts remains somewhat limited. This is because older adults in rural areas often face structural constraints, including limited access to healthcare resources, uneven provision of public services, and weak social support networks, meaning that some indicators in standardized instruments may not fully reflect their actual wellbeing (12). As population aging continues, the limitations of single-dimensional measurement approaches are becoming increasingly evident, and the academic community is gradually shifting toward more comprehensive, multi-dimensional frameworks for assessing the wellbeing of older adults.

Against this backdrop, the World Health Organization introduced the framework of active aging in 2002, which has since become a key theoretical foundation for shifting research on the wellbeing of older adults. This framework emphasizes that older people are active agents capable of continuing to participate in social development and realizing their full potential. It is built on three core pillars, namely, health, participation, and security, and advocates for the optimization of institutional frameworks and social support systems to enable older adults to maintain a high quality of life throughout the life course. The framework of active aging places greater emphasis on older adults’ developmental capacity, social integration, and agency than traditional aging research, which emphasizes passive social protection. The WHO Framework on Active Aging primarily provides a theoretical and policy-oriented perspective rather than constituting a specific standardized measurement system. The AAI is a multidimensional measurement framework developed in accordance with the broader concept of active aging, with its own domain structure and set of indicators. Although the AAI is conceptually related to the WHO Framework on Active Aging, its domains do not correspond directly or one-to-one with the three pillars of “health, participation, and security.” Therefore, it should not be regarded as a direct operational measurement tool for the WHO framework. In recent years, the academic community has further expanded wellbeing measurement within the AAI framework. For instance, research based on the expected utility framework has incorporated factors such as consumption, health, mortality, and wealth into a unified analytical system (14), significantly enhancing the explanatory power and robustness of wellbeing measurement.

As measurement methods improved, the focus of research has gradually shifted from “how to scientifically measure wellbeing” to identifying its influencing factors and clarifying the underlying mechanisms. Previous studies have shown that the development and evolution of wellbeing among older adults in rural areas result from the combined effect of multiple factors, including the economic base, healthcare provision, and institutional environment (15). In particular, the improvement and upgrading of rural infrastructure can effectively meet the basic subsistence and developmental needs of rural residents and thus serves as a key external factor underpinning the wellbeing of older adults (16). There is a significant positive association between healthcare conditions and rural wellbeing, while the unequal distribution of healthcare resources not only directly undermines the health rights of older adults but also weakens social cohesion and political stability, thereby hindering improvements in overall human wellbeing (17, 18). Furthermore, community environmental characteristics, the provision of public services, and the development of pension security systems constitute a crucial institutional environment that shapes residents’ wellbeing (19).

However, most of the aforementioned studies have primarily focused on traditional economic and institutional variables, with digitalization as an emerging structural factor receiving relatively limited attention. Digital technologies are currently permeating rural communities at an unprecedented pace and scale. For older adults in rural areas, rural digitalization has the potential to enhance their wellbeing by expanding access to information and improving access to telemedicine and older care services; however, it may also exacerbate inequalities in welfare access due to barriers such as the digital divide, limited digital skills, and psychological resistance. Therefore, within the theoretical framework of active aging, digitalization should not be viewed merely as a technological tool but rather as a key external mechanism for empowering rural older adults by strengthening their health security, social participation, and development capacity.

Although some studies have begun to examine the impact of digital technologies on the quality of life and subjective wellbeing of older adults (20), in the Chinese context, recent studies have also addressed the age-related digital divide, community-based digital divides, digital support for older adults, and the impact of rural digitization on the health of older adults (7–10). While these studies have enriched our understanding of the relationship between digital technology and health and quality of life, their outcome variables remain focused on specific dimensions, such as physical or mental health. In contrast, further research that integrates health security, social participation, living conditions, and the development environment into a unified, multidimensional wellbeing framework is needed. Such an approach would enable a more comprehensive examination of the relationship between rural digitalization development and the rural older overall wellbeing within the framework of active aging. This gap provides a clear entry point for systematically examining the impact of rural digitalization on the wellbeing of older adults in rural areas from the perspective of active aging.

### 2.2 Multidimensional linkages between rural digitalization and the wellbeing of older adults in rural areas

As the digital economy expands into rural areas, rural digitalization is gradually becoming a key driver of rural revitalization. Existing research generally indicates that rural digitalization can profoundly influence the quality of life and overall wellbeing of older adults in rural communities by improving access to information, optimizing the provision of public services, and expanding channels for social participation (5). However, this relationship is not simply linear but rather a complex process shaped by multiple interacting socio-economic mechanisms that influence both quality of life and developmental capacity. Overall, existing studies indicate that the positive effects of digitalization on the wellbeing of older adults in rural areas are primarily reflected across multiple dimensions, including access to practical services, psychosocial wellbeing, and economic participation.

The application of digital technologies, such as “Internet Plus Healthcare,” has significantly improved the accessibility and convenience of healthcare services for older adults in rural areas. This model provides new channels for accessing medical resources and has a positive effect on improving health behaviors and overall wellbeing (21). In addition, public service platforms, such as e-government services and online payment systems, have greatly enhanced the convenience of daily life for older adults in rural communities. At the socio-psychological level, the widespread adoption of the internet has provided rural older adults with abundant opportunities for cultural and recreational engagement. Through online communication, older people can maintain social connections with family and friends, strengthen their sense of family belonging, and receive social support, thereby alleviating feelings of loneliness (22). This emotional benefit derived from social networking represents an important psychological mechanism through which digital technology influences the wellbeing of older adults. From an economic perspective, the development of the digital economy has further driven the growth of the “silver economy,” significantly increasing consumption levels and optimizing consumption patterns among older rural residents (23). Online shopping and digital financial services not only provide older adults with broader consumer choices but also enhance their capacity to participate in market activities.

The benefits of digital empowerment are constrained by factors such as digital access, educational attainment, physical health, and social support. For some older adults in rural areas, complex digital platforms, the rapid pace of technological change, and the increasing shift from offline to online service provision may, to some extent, raise barriers to digital inclusion (24). Recent Chinese studies have further investigated variations in the digital divide among older adults from multiple perspectives, including age and socioeconomic differences (7), as well as urban–rural, gender, and health disparities (8). Additionally, research has explored the relationships between the digital divide, digital support, and health-related quality of life among rural older adults (9), and has expanded to examine the link between rural digitalization and health outcomes (10). Moreover, the impact of digitalization on social relationships and social trust is complex: while digital technologies can expand social networks and facilitate information exchange among older adults, disparities in digital literacy across groups may also lead to significant inequalities in social participation outcomes (25). Therefore, digitalization does not automatically translate into improved wellbeing because its effects may vary considerably depending on individual characteristics and regional contexts.

Although existing research has provided preliminary insights into the impact of rural digitalization on the wellbeing of older adults in rural areas from the perspectives of practical services, psychosocial factors, and economic participation, research on the digital divide, rural digitization, and the health of older adults in China is continually expanding; however, existing studies still tend to focus primarily on individual-level digital access and usage or specific health outcomes. This study examines provincial-level rural digital development, encompassing digital infrastructure and the digitization of the rural economy, daily life, governance, and ecosystem, and its relationship with the comprehensive wellbeing of the rural older adults. Given the differences in measurement levels and conceptual scope, further analysis of the potential mediating mechanisms underlying this relationship, including self-efficacy, social networks, and social trust, is warranted.

### 2.3 Evolution of identification strategies in research on digitalization and the wellbeing of older adults

In recent years, empirical research on the wellbeing of older adults in rural areas has developed a multivariate analytical framework grounded on traditional econometric models, primarily relying on classical methods such as multiple regression (e.g., ordinary least squares (OLS) models) and propensity score matching (PSM). These approaches have made important contributions to the control of confounding factors, selection bias, and partial causal relationships. For instance, existing studies have used ordered probit models to estimate the marginal effects of key variables across different levels of wellbeing, while employing OLS regression as a robustness check, thereby establishing a dual-validation framework for identifying underlying associations (26). By employing a PSM model to account for differences in characteristics between the treatment and control groups, estimation bias can be effectively reduced, thereby enabling a more accurate identification of the net effect of digital literacy on the wellbeing of older adults in rural areas (27).

As research advances, addressing only observable selection bias is no longer sufficient for identifying causal relationships, as unobserved individual heterogeneity, non-linear relationships among variables, and heterogeneous treatment effects have become important challenges. To address time-invariant individual differences, fixed-effects (FE) models have been introduced. By controlling for both individual and time FEs, these models allow for more accurate estimation of the long-term dynamic effects of factors such as rural digitalization development on the wellbeing of older adults (28). In addition, to capture the non-linear nature of digitalization effects, threshold regression models have been applied. These models can identify structural changes in the impact of digitalization on the wellbeing of older adults when a key variable crosses a specific “threshold,” thereby revealing the limiting conditions of digital literacy in shaping the wellbeing of rural older adults (29). Furthermore, to clarify the independent contributions of different factors in complex contexts, studies have applied both linear and logistic regression models to assess the effects of environmental perception on wellbeing, while also using hierarchical regression to sequentially incorporate variables from different dimensions, thereby isolating the unique contribution of each factor (30).

Although traditional econometric models and multivariate methodological frameworks have demonstrated strong applicability in identifying correlations, testing causality, and analyzing mechanisms, they still have several limitations. First, at the level of variable measurement, OLS models struggle to capture the ordinal nature of ordered dependent variables, which may lead to estimation bias, whereas ordered Probit models rely on strict distributional assumptions, although they are more consistent with such data structures, and their estimates can be sensitive to outliers. Second, at the causal identification level, the performance of PSM models depends heavily on the completeness of observed covariates; the omission of key confounding variables may result in biased estimates. Third, in terms of capturing non-linearity and heterogeneity, fixed-effects models are unable to identify non-linear threshold effects between variables, while threshold regression models are sensitive to outliers in estimating threshold values, although they are capable of capturing structural changes. Furthermore, when handling multidimensional environmental variables, both linear and logistic regression models often suffer from multicollinearity, which may weaken the reliability and validity of parameter estimates.

In summary, existing research has three main limitations: first, it often treats digital technology as a single instrumental variable, overlooking its broader role as part of a “technology-institution-economy” composite system. Second, it does not sufficiently explain the mechanism of action and heterogeneous effects through which rural digitalization influences the wellbeing of older adults in rural areas. Third, traditional econometric models face difficulties in effectively addressing issues such as ordered response bias, omitted variable bias, and non-linearity. This study conducts a systematic analysis of how rural digitalization affects the wellbeing of older adults in rural areas, as well as its mechanism of action and heterogeneous effects.

## 3 Theoretical analysis and research hypotheses

### 3.1 Rural digitalization and the wellbeing of rural older adults

Against the backdrop of active aging, digital development is profoundly transforming the allocation of resources, the delivery of public services, and the structure of social governance in rural areas. It is gradually becoming a crucial external institutional factor influencing the wellbeing of rural older adults. Existing research indicates that digital technologies can enhance the quality of life of older adults by improving access to information, enhancing access to public services, and expanding opportunities for social participation (31). Digital infrastructure, the digital economy, digital governance, and digital public services are steadily extending into rural areas as the development of digital villages continues to advance, providing new channels for rural older residents to access healthcare, social security, public information, and social support (32, 33). By expanding access to healthcare, social connections, and public services, rural digitalization may contribute to multiple dimensions of older adults’ wellbeing, particularly those related to health, social participation, and security emphasized in the active aging framework. However, rural digitalization may also produce a “crowding-out effect.” Due to disparities in digital skills, information literacy, and technological adaptability among rural older adults, some may be excluded from the digital society and struggle to adapt to the digital environment, thereby contributing to a new form of digital divide (34). Existing research indicates that digital resources tend to be concentrated among groups with stronger technical capabilities or greater resource endowments, and this “Matthew effect” may further widen welfare disparities between different groups (35). Furthermore, technology anxiety, digital exclusion, and the psychological dimension of the digital divide may also undermine the social participation and sense of digital inclusion of older adults (36). Consequently, rural digitalization may exert dual effects on the wellbeing of rural older adults, generating both gains and forms of exclusion.

Overall, the impact of rural digitalization on the wellbeing of older adults in rural areas remains predominantly positive. On the one hand, digitalization can enhance rural residents’ access to information and improve resource allocation efficiency, while also increasing access to public services such as healthcare and social security, thereby improving the quality of life and health status of older adults (37). On the other hand, the development of digital governance and digital public services contributes to improving grassroots governance efficiency, thereby enhancing the sense of security, social integration, life satisfaction and opportunities for social participation of older adults (33). Although rural digitalization may entail certain risks of digital exclusion, it can generally promote the wellbeing of older adults in rural areas. Based on this, the following research hypothesis is proposed:

*H1*: Rural digitalization enhances the wellbeing of older adults in rural areas.

### 3.2 The mechanisms underlying social trust

Against the backdrop of ongoing efforts to promote active aging and rural digitalization, the wellbeing of older adults in rural areas has received increasing scholarly attention. Existing research indicates that the wellbeing of older people is influenced not only by income and public service provision but also by perceptions of social relationships and levels of social trust (38). Social trust reflects the extent to which individuals trust others, social organizations, and the institutional environment. Higher levels of social trust can enhance the sense of social security, belonging, and willingness of older adults to participate in social life, thereby improving their life satisfaction and overall wellbeing (39). Rural digitalization is profoundly reshaping information dissemination, social interaction patterns, and the delivery of public services in rural areas. Therefore, integrating “rural digitalization -social trust-wellbeing of older adults in rural areas” into a unified analytical framework is of great significance for clarifying the mechanisms through which digitalization enhances the wellbeing of older adults in rural contexts.

Rural digitalization development can, to a certain extent, enhance social trust among older adults in rural areas. On the one hand, improvements in rural digitalization have strengthened access to information and public services in rural communities. With the increasing prevalence of the internet, e-government services, and online public platforms, rural residents can obtain information on policies, healthcare, and social security more conveniently, thereby strengthening their recognition of grassroots governance capacity and the public service system (33). Meanwhile, the development of digital rural governance has strengthened interactions among residents and between residents and local governments. Digital platforms, WeChat groups, and online village affairs disclosure systems have improved information transparency and communication efficiency, thereby enhancing residents’ trust in government governance and the broader social environment (40). Existing research further indicates that digital participation can increase the political trust and social identification levels of residents (41). The preceding discussion aims to elucidate the indirect pathways through which rural digitalization may shape the social trust of older adults, rather than assuming that older adults are necessarily active or proficient users of digital technologies. This distinction is consistent with the analytical framework of this study, which treats the regional digital environment as the exposure variable rather than individual-level digital behavior or technology.

Furthermore, higher levels of social trust can positively influence the wellbeing of rural older adults. Social trust encourages older residents to actively participate in social activities and community interactions, thereby strengthening their access to social support and improving both social participation and life satisfaction (20). Simultaneously, it can reduce uncertainty and psychological stress during social interactions, enhancing their sense of social security and overall wellbeing (38). In the context of digitalization, when older adults have greater trust in social relationships, grassroots governance, and public service systems, they are better able to integrate into the digital society and access a wider range of life resources and public service support through digital channels, ultimately improving their overall wellbeing (31).

However, digital development may also generate a certain “crowding-out effect.” Due to insufficient digital skills and limited information literacy, some older adults in rural areas may experience anxiety, alienation, and even distrust when confronted with complex digital platforms and online information (42). If the pace of digitalization exceeds the adaptive capacity of older populations, it may weaken their willingness to participate in social activities and further undermine social trust (43). Therefore, the influence of rural digitalization on social trust and, consequently, on the wellbeing of older adults is somewhat ambivalent; however, the overall positive effect is still considered to be dominant. Based on the above analysis, the following research hypothesis is proposed:

*H2*: Rural digitalization may be associated with the enhancement of wellbeing of older adults in rural areas by increasing their level of social trust.

### 3.3 The mechanism of self-efficacy

The wellbeing of older adults in rural areas is influenced not only by external factors, such as economic conditions and public service provision, but also by individual psychological cognition and subjective agency. Older adults are not merely passive recipients of external resources; rather, they can actively shape their own wellbeing through cognitive processes and behavioral choices. Self-efficacy reflects an individual’s subjective judgment of their ability to perform specific actions or cope with particular situations. Higher levels of self-efficacy can enhance the adaptability, willingness to participate in social life, and life satisfaction of older adults, thereby improving their overall wellbeing (44, 45). Rural digitalization is profoundly reshaping how rural communities access information, engage in social interactions, and obtain public services. Therefore, integrating the relationship between “rural digitalization, self-efficacy, and wellbeing of older adults in rural areas” into a unified analytical framework is of great significance for revealing the underlying mechanisms through which digitalization improves the wellbeing of older adults in rural areas.

To a certain extent, digital development in rural areas can enhance the self-efficacy of older adults in rural communities. Improvements in regional digital infrastructure and services may expand the access of older adults to health information, public services, and social security resources, thereby potentially enhancing their sense of control over their daily lives and surrounding environment (46, 47). At the individual level, greater familiarity with digital technologies may reduce technology-related anxiety and barriers to use, potentially improving digital adaptability and behavioral confidence. However, within the framework of this study, these individual-level processes are considered potential pathways through which a more developed regional digital environment may influence self-efficacy, rather than the effects directly measured by the Rural digitalization Index (36, 48). Existing research further indicates that digital engagement can enhance the self-perception and perceived competence of older adults (29). Individual-level digital engagement may constitute a potential pathway through which improvements in the regional digital environment influence older adults’ self-efficacy.

Furthermore, higher self-efficacy can significantly enhance the wellbeing of rural older adults. Stronger self-efficacy increases the willingness of older adults to participate in social life and strengthens their sense of control over daily activities, thereby improving their access to social support and overall life satisfaction (49). Simultaneously, self-efficacy can reduce anxiety and feelings of helplessness, enhancing the capacity of older adults to cope with health, financial, and family-related pressures (50). In the context of digitalization, older adults with higher self-efficacy are better able to adapt to the digital society and proactively use digital resources to improve their quality of life, thereby further enhancing their overall wellbeing (44).

However, digital development may also generate a certain “crowding-out effect.” Due to insufficient digital skills or high learning costs, some older adults in rural areas may experience technological anxiety and self-doubt when confronted with complex digital platforms (43). If the pace of digitalization exceeds the adaptive capacity of older populations, it may undermine their confidence in digital participation and further reduce their self-efficacy (51). Therefore, the impact of rural digitalization on self-efficacy and, consequently, on the wellbeing of older adults is somewhat ambivalent; however, the overall positive effect is still considered dominant. Based on the above analysis, the following research hypothesis is proposed:

*H3*: Rural digitalization may be associated with the enhancement of wellbeing of older adults in rural areas by enhancing their sense of self-efficacy.

### 3.4 The mechanism of social networks

Against the backdrop of ongoing efforts to promote active aging and rural digitalization, the wellbeing of older adults in rural areas is influenced not only by external factors, such as economic resources and public service provision, but also by the extent of their social embeddedness and the quality of their social interactions. Existing research indicates that social networks serve as a crucial channel through which individuals access information, emotional support, and social recognition. Strong social networks enhance the social connections and emotional support of older adults, thereby improving their life satisfaction and overall wellbeing (52). Rural digitalization is profoundly reshaping information dissemination patterns and modes of social interaction in rural communities. Therefore, integrating the framework of “rural digitalization–social networks–wellbeing of older adults in rural areas” into a unified analytical model is of great significance for elucidating the social mechanisms through which digitalization enhances the wellbeing of older adults in rural contexts.

Digital development in rural areas can expand the social networks of older adults in rural communities to a certain extent. The internet and mobile communication technologies have broken down geographical barriers, enabling older adults to stay in touch with family members and friends in other locations more easily, thereby strengthening family bonds and daily interactions (53). Digital platforms and social media also provide new avenues for social participation, allowing older adults to broaden their social connections and increase the frequency of interaction through online communication, community engagement, and information sharing (54). Existing research further indicates that an improved digital environment can promote social connections and the accumulation of social capital among older adults (55).

Furthermore, stronger social networks can positively influence the wellbeing of older adults in rural areas. Robust social ties not only provide continuous emotional support, alleviate feelings of loneliness and social isolation, and thereby improve mental health and life satisfaction (56), but also facilitate access to information and social support, enhancing the ability of older adults to cope with life risks and uncertainties. In rural contexts, stable social networks enable older residents to receive stronger family and community support, thereby improving their overall wellbeing (57, 58).

However, digital development may also generate a certain “crowding-out effect.” Due to insufficient digital skills or limited technological adaptability, some older adults in rural areas may face communication barriers and an increased risk of social isolation during digital social interactions (43). If the pace of digitalization exceeds the adaptive capacity of older populations, it may weaken their willingness to engage in offline social interactions and community participation, thereby constraining their social network expansion (24). Therefore, the impact of rural digitalization on social networks and, consequently, on the wellbeing of older adults is somewhat ambivalent; however, the overall positive effect remains dominant. Based on the above analysis, the following research hypothesis is proposed:

*H4*: Rural digitalization may be associated with the enhancement of wellbeing of older adults in rural areas by expanding their social networks.

The theoretical framework and mechanisms of this study are illustrated in Figure 1.

**Figure 1:**
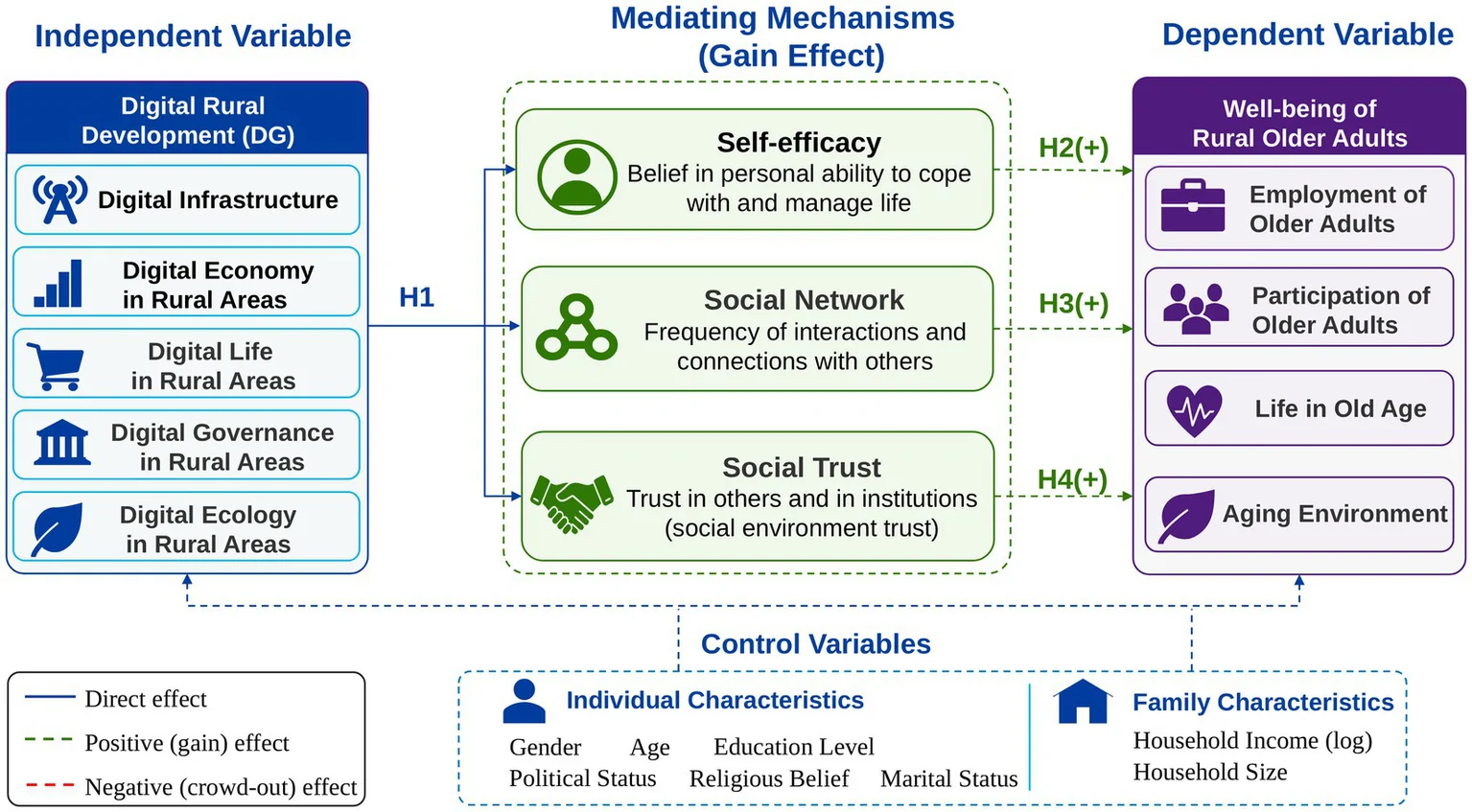
Schematic diagram.

## 4 Materials and methods

### 4.1 Data source

Data on rural digital development were obtained from the China Taobao Village Research Report, the Peking University Digital Inclusive Finance Development Index Report, the Zhejiang University Carter Center for Agricultural and Rural Development China Agriculture Research Database, various statistical yearbooks, and the Economy Prediction System (EPS) Global Statistics Platform. Data on the wellbeing of older adults in rural areas, institutional variables, and control variables were sourced from the China Family Panel Studies (CFPS). The CFPS conducted its baseline survey in 2010, followed by a biennial follow-up survey. As questions on Parent–Child relationships were introduced only in 2016, this study uses data from waves 2016, 2018, 2020, and 2022.

To enhance the transparency of the sample construction process, this study first uses the official CFPS individual tracking identifier (PID) to identify repeated observations of the same rural older resident across different survey years. The combination of PID and survey year (Year) serves as a unique identifier for individual-year observations. The verification confirmed that each PID corresponds to only one valid observation per survey year. Based on this procedure, the study population is limited to rural residents aged 60 and older, and eligible CFPS individual samples are matched with provincial-level rural digital development data.

To address missing observations for certain years or provinces within the provincial rural digital development indicators, this study fills gaps while preserving the time-series characteristics and interprovincial differences in the original indicators. For indicators with continuous historical observations and relatively stable trends over time, trend extrapolation is used to estimate values for missing years that fall at the boundaries of the sample period, based on the observed trends within the respective provinces. For missing years that fall between two observed years, linear interpolation is used to estimate the corresponding values based on the observations from the immediately preceding and following years. Missing-value handling is conducted at the provincial level and does not involve CFPS individual-level survey variables or alter the inclusion or exclusion criteria for individual samples. After imputing the provincial-level indicators, the rural digital development data are matched with the CFPS microdata based on the province and survey year.

Following sample screening and data matching, the final sample comprised 7,760 individual-year observations, corresponding to 4,241 independent rural older residents across 24 provincial-level administrative units; samples from Beijing were excluded due to extensive missing data. Consequently, the data constitute an unbalanced longitudinal panel rather than a balanced panel in which all individuals participated in all four rounds of the survey. Regarding longitudinal tracking, the 2016 baseline sample included 2,393 independent older residents, of whom 1,424 were also observed in 2018, 647 were continuously observed in both 2018 and 2020, and 337 had valid observations across all four survey rounds (2016, 2018, 2020, and 2022). Using the 2016 sample as the baseline, the retention rate for continuous follow-up through 2018 was 59.51%, that through 2020 was 27.04%, and that for respondents completing all four rounds through 2022 was 14.09%. Note that samples across survey waves do not necessarily comprise the same cohort; therefore, this study treats the data as an unbalanced longitudinal panel, retaining valid individual-year observations that meet the sample selection criteria without requiring individuals to participate in all four survey waves.

### 4.2 Model setup

Given the high dimensionality and complexity of factors influencing the wellbeing of older adults in rural areas (59, 60), traditional linear regression models are prone to specification bias and omitted variable problems. Therefore, this study adopts a double machine learning framework. By integrating regularization techniques and machine learning algorithms, the model automatically selects relevant variables from high-dimensional controls and reduces estimation bias, thereby improving the robustness of the estimated effects of the core explanatory variables. The baseline model is specified as follows:

$$WB_{\mathrm{it}}=\theta_{0}DG_{\mathrm{it}}+g_{0}(T_{\mathrm{it}})+U_{\mathrm{it}},E(U_{\mathrm{it}}|T_{\mathrm{it}},D_{\mathrm{it}})=0$$ (1)

In Equation 1, $WB_{\mathrm{it}}$ represents the wellbeing of older adults in rural areas, and the latter denotes the level of digital development in rural areas. $\theta_{0}$ is the coefficient of interest in this study, i represents the province, and t represents time. $T_{\mathrm{it}}$ is a set of control variables, $g_{0}(\cdot )$ is an unknown nonlinear function that captures the effect of the independent variables on the dependent variable. $U_{\mathrm{it}}$ and $v_{\mathrm{it}}$ are disturbance terms with an expected value of zero.

Simultaneously, the following auxiliary equation is specified to model the effect of control variables on the core explanatory variables:

$$DG_{\mathrm{it}}=m_{0}(T_{\mathrm{it}})+v_{\mathrm{it}},E[v_{\mathrm{it}}|T_{\mathrm{it}}]=0$$ (2)

In Equation 2, $m_{0}(\cdot )$ represents the nonlinear effect function of the control variables on the level of digital development, and $v_{\mathrm{it}}$ is the error term. To address potential endogeneity issues, such as reverse causality and omitted variables, this study further constructs a partial linear instrumental variable model based on the double machine learning framework as an extension of the baseline model, as specified in Equations 3, 4:

$$WB_{\mathrm{it}}=a_{1}DG_{\mathrm{it}}+g(\text{Control}s_{\mathrm{it}})+U_{\mathrm{it}}$$ (3)

$$Z_{\mathrm{it}}=m(\text{Control}s_{\mathrm{it}})+V_{\mathrm{it}}$$ (4)

Here, $Z_{\mathrm{it}}$ serves as an instrumental variable for the primary explanatory variable $DG_{\mathrm{it}}.$ $\text{Control}s_{\mathrm{it}}$ denotes the set of control variables, while g(·) and m(·) represent the unknown nonlinear functions that capture the effects of the control variables on the dependent and instrumental variables, respectively.

Based on Equation 1, the full sample is divided into a main sample of size n and an auxiliary sample of size N–n. Using the auxiliary sample to obtain ${\hat{g}}_{0}$ and related nuisance parameters, the main sample is then used to construct the final estimate $\hat{\theta }$ , as specified in Equation 5:

$${\hat{\theta }}_{0}={\left(\frac{1}{n}\sum_{i\in I,t\in T}DG_{\mathrm{it}}^{2}\right)}^{-1}\frac{1}{n}\sum_{i\in I,t\in T}DG_{\mathrm{it}}[WB_{\mathrm{it}}-{\hat{g}}_{0}(T_{\mathrm{it}})]$$ (5)

Furthermore, based on the estimated value of $g_{0}$ , this study constructs an unbiased estimator for the main sample, denoted as $\theta_{0}$ , as specified in Equation 6:

$${\hat{\theta }}_{0}={\left(\frac{1}{n}\sum_{i\in I,t\in T}{\hat{V}}_{\mathrm{it}}DG_{\mathrm{it}}\right)}^{-1}\frac{1}{n}\sum_{i\in I,i\in T}{\hat{V}}_{\mathrm{it}}[WB_{\mathrm{it}}-{\hat{g}}_{0}(T)]$$ (6)

In summary, the double machine learning approach uses machine learning methods to model high-dimensional control variables nonparametrically. By combining orthogonalization with cross-fitting (cross-validation) techniques, model specification bias and potential endogeneity problems are reduced, thereby improving the robustness of the estimated effects of the core explanatory variables. Given the multi-wave panel structure of the China Family Panel Studies (CFPS), in which respondents are observed across multiple years, we conduct a supplementary analysis using individual and year fixed effects. We apply a within-individual mean-differencing transformation to the dependent variable, core explanatory variables, and time-varying controls. This approach eliminates time-invariant unobservable heterogeneity at the individual level, ensuring that the estimates rely primarily on within-respondent variation over time.

Regarding statistical inference, the baseline model retains provincial and annual fixed effects. To address repeated observations and common provincial shocks, we employ cluster-robust standard errors at the provincial level. Additionally, because observations from the same respondent across different years may exhibit correlated errors, we report cluster-robust standard errors at the individual level as a supplementary sensitivity check. The roles of these specifications must be distinguished: individual fixed effects control for time-invariant individual heterogeneity; provincial clustering accounts for error correlation arising from common shocks within the same province; and individual clustering allows for correlation in the disturbance terms of the same respondent across different years.

### 4.3 Variable definition

#### 4.3.1 Dependent variable: wellbeing of older adults in rural areas

Existing research on measuring the wellbeing of older adults can be broadly divided into two categories: unidimensional indicators and multidimensional comprehensive evaluation frameworks. The former focuses on single dimensions, such as income, health, or life satisfaction, while the latter, grounded in the theory of active aging, constructs multidimensional systems encompassing health, participation, and security. Among these frameworks, AAI is one of the most representative multidimensional measurement frameworks. At the theoretical level, this study adopts the WHO’s Active Aging Framework as the foundation for understanding the multidimensional nature of wellbeing among rural older adults. At the measurement level, it draws on the AAI’s approach to constructing multidimensional indicators. Notably, the AAI is not a direct measurement of the WHO’s three pillars of “health, participation, and security”; rather, it is a multidimensional framework with its own domain structure and set of indicators. Given that the original AAI indicator system was primarily based on the European social context and paid insufficient attention to the specific characteristics of older adults in rural China, particularly in relation to digital inclusion, intergenerational family relationships, and local social support (5). This paper examines the characteristics of China’s rural aging population, the current state of rural digital development, and data availability to localize and expand relevant indicators. This study conceptualizes the wellbeing of older people in rural areas as a multidimensional and comprehensive construct grounded in the theory of active aging, rather than as a single subjective assessment or health outcome. Wellbeing encompasses not only older adults’ health and living conditions but also their ability to participate in family and social activities, remain economically active, secure their livelihoods, and access external social support and environmental resources. The World Health Organization’s active aging framework emphasizes that maintaining a good quality of life in later life requires comprehensive support in health, participation, and security, as well as being shaped by social, economic, and physical environments. Accordingly, this study does not equate wellbeing solely with subjective wellbeing or life satisfaction; instead, it incorporates older adults’ actual living conditions, opportunities for participation, and access to social and environmental support within a unified multidimensional evaluation framework.

Building on this foundation, this study, drawing on AAI’s multidimensional measurement approach, has adapted and expanded the relevant indicators to suit the local context to better reflect the realities of rural China. The wellbeing of older adults in rural areas is categorized into four core dimensions: employment, participation, life in old age, and the aging environment. To develop a comprehensive measure of wellbeing, this study first derives wellbeing-related indicators from micro-level survey data on rural older adults to construct a set of basic wellbeing indicators. These indicators are then integrated using the entropy-weighted technique for order preference by similarity to the ideal solution and aggregated into a comprehensive wellbeing index for rural older adults. The specific evaluation indicators are presented in Table 1.

**Table 1:** Evaluation indicator system for the wellbeing of older adults in rural areas.

Primary indicators	Secondary indicators	Specific indicators	Indicator properties
Employment of older adults	Employment rates among older adults	Employment rates among older adults	+
Participation of older adults	Level of involvement in family care	Doing housework or taking care of children	+
		Taking care of household chores or looking after parents	+
	Level of political participation	Older adults who did or did not vote in the election	+
	Level of participation in volunteer activities	Whether to participate in charitable giving	+
		Is or is not a union member	+
Life in old age	Health concepts	Exercise habits	+
	Level of physical health	Physical health over the past 2 weeks	+
	Physical health resources	Older adults’ self-reported health status	+
	Quality of life	Self-assessed life satisfaction	+
	Quality of spiritual life	Frequency of visits with children	+
	Mental health resources	Mental health of older adults	+
	Income level	Self-Assessment of Income Adequacy	+
	Awareness of active learning	Whether you read regularly	+
	Level of material abundance	Whether or not you have basic old-age insurance and basic Health Insurance	+
Aging environment	Level of technical support	Internet Usage	+
		High school graduate or higher	+
	Level of social skills	Frequency of contact with family and friends	+
	Social environment	Satisfaction with the government	+

Furthermore, the concept of “wellbeing” adopted in this study is not entirely synonymous with subjective wellbeing or quality of life. Subjective wellbeing primarily reflects an individual’s subjective evaluations of their lives, including life satisfaction and positive or negative affect, whereas quality of life typically encompasses physical health, mental wellbeing, social relationships, and daily functioning. In contrast, this study adopts an active aging perspective and incorporates the health and living conditions, productive activities, social participation, livelihood security, and external support environment of older adults in rural areas into a unified evaluation framework. Accordingly, the wellbeing index developed in this study is not intended to replace single measures of subjective wellbeing or quality of life; rather, it aims to provide a comprehensive assessment of the overall wellbeing of rural older adults based on multidimensional indicators of their objective living conditions, participation, and access to resources and support.

##### 4.3.1.1 Employment of older adults

From the perspective of active aging, employment is an important means through which older adults can engage in productive activities and is therefore closely linked to the “participation” dimension of wellbeing. As a core dimension of the rural older adult’s wellbeing index, older adults’ employment captures the productive labor participation of older adults. Previous research indicates that meaningful and productive activities, such as employment, have a significantly greater impact on the physical and mental health of older adults than obligatory tasks, such as housework. Compared with retirement or unemployment, continued employment helps older adults build cognitive reserves, thereby enhancing cognitive performance and delaying cognitive decline (61).

##### 4.3.1.2 Participation of older adults

This dimension refers to non-productive activities undertaken by older adults, such as volunteer work, political participation, and household chores. Research indicates that such activities can help activate the human capital of older adults through two pathways: enhancing cognitive functioning (62) and improving mental health (63). This study retained three indicators from the AAI-EU framework, namely, participation in volunteer activities, family caregiving, and political elections, to measure the level of participation among older adults. This dimension corresponds directly to the “participation” component of the active aging framework, reflecting the extent to which older adults continue to participate in family, community, and social activities and maintain meaningful social roles.

##### 4.3.1.3 Life in old age

Within the active aging framework, this dimension primarily reflects the foundations of health and security that enable older adults to maintain a good quality of life in later life. Independence, health, and safety are three key characteristics of later life that serve as prerequisites for older adults to engage in employment and participate in social activities. Grounded in the concepts of “security” and “health” within active aging theory and drawing on the EU Active Aging Index framework and relevant studies (64), this study selects indicators across multiple dimensions, including physical activity, cognitive and psychosocial wellbeing, functional capacity, access to learning opportunities, and livelihood security, to comprehensively assess the living conditions of older adults. By incorporating both health-related functioning and livelihood security, this dimension emphasizes that the wellbeing of older adults depends not only on maintaining physical and cognitive functioning but also on access to the resources and opportunities necessary to live independently and securely.

##### 4.3.1.4 The aging environment

The aging environment primarily reflects the external social, technological, and institutional conditions that support older adults in maintaining their health, participating in social life, and securing their livelihood. This dimension does not measure individual skills or digital literacy; rather, it emphasizes the external resources and support available to them, including social networks, public services, access to technology, and other environmental conditions relevant to wellbeing in later life (65, 66).

These four dimensions represent a contextualized application of the core concepts of active aging, considering the socio-economic environment, family structures, and data availability among older adults in rural China. Collectively, these dimensions provide a comprehensive framework for assessing wellbeing, encompassing both living conditions and access to external resources and support.

With the rapid increase in the proportion of older internet users, enhancing their ability to use smart technologies has become an effective way to improve their wellbeing in the information age. Social support serves as a protective factor for mental health, particularly in rural areas where children often migrate for work, leaving older parents to live alone. Strong social support helps older adults develop a positive self-image. Related studies have found that the interaction between social support and intrinsic capacity significantly influences the trajectory of functional ability maintenance among older adults (67). This indicates that improving the social environment is a crucial strategy for meeting the needs of older adults for health maintenance and social participation in the context of actively addressing population aging.

#### 4.3.2 Explanatory variable: level of rural digital development

This study draws on policy documents such as the Guidelines for Digital Rural Development 2.0 and the classification standards of the indicator system in the County-Level Digital Rural Index compiled by the Institute for New Rural Development at Peking University. Building on existing research (29) and following the principle of data availability, we construct an indicator system for rural digital development across five dimensions: digital infrastructure, rural economy digitalization, rural life digitalization, rural governance digitalization, and rural ecosystem digitalization. The specific indicators are presented in Table 2.

**Table 2:** Evaluation indicator system for rural digitalization.

Primary indicators	Secondary indicators	Specific indicators
Digital Infrastructure in Rural Areas	Network Infrastructure	Rural broadband subscribers (Ten thousand households, +)
		Average number of computers per 100 rural households at year-end (unit, +)
		Average number of mobile phones per 100 rural households at year-end (unit, +)
	Information Services Infrastructure	Percentage of Taobao Villages among all administrative villages (%, +)
	Digital Financial Infrastructure	Coverage of digital financial infrastructure (−, +)
		Depth of digital financial infrastructure adoption (−, +)
Digitalization of the Rural Economy	Level of digital production	National modern agriculture demonstration projects (unit, +)
		Number of digital agriculture companies (unit, +)
	Level of digital finance	Level of digitalization in inclusive finance (−, +)
	Level of digital marketing	E-commerce sales (in billions of yuan, +)
		Percentage of enterprises engaged in e-commerce transactions (%, +)
	Level of supply chain digitization	Average number of deliveries per week in rural areas (times, +)
		Rural delivery routes (km, +)
Digitization of Rural Life	Level of development of internet culture	Percentage of households with cable TV subscriptions (%, +)
	Digital consumption levels among farmers	Per capita expenditure on transportation and communications by rural households (yuan, +)
	Level of digital education	Percentage of multimedia classrooms in rural primary and secondary schools (%, +)
		Number of online education companies (unit, +)
Digitalization of Rural Governance	Internet + Government Services	Number of decisions issued by agricultural and rural affairs departments (unit, +)
	Level of smart emergency management	Total number of seismic stations (units, +)
		Number of automatic weather stations (units, +)
Rural Ecological Digitization	Green living environment	Percentage of mechanized road sweeping (%, +)
	Level of green production	Number of water-saving irrigation machines (thousands of units, +)
		Total pesticide application in mechanized agricultural production (10,000 metric tons, −)

Notably, all the aforementioned indicators are macro-level structural variables measured at the provincial level. They reflect the overall digital environment in the regions where rural residents live, rather than the digital skills, literacy, or technology-use behaviors of individual rural older adults. This distinction carries significant theoretical implications: under an external structural condition, the regional digital environment can influence older adults’ wellbeing without requiring them to personally use digital technologies. Even if older adults are not active digital users, improvements in the digital environment may still positively impact their wellbeing through indirect channels, such as enhancing access to public services, strengthening community connectivity, and improving governance efficiency. Therefore, this study’s discussion of digital skill enhancement, Internet use, and digital participation serves solely as a theoretical explanation of possible mediating pathways, rather than a direct measurement of individual-level digital competence or an attribution of effects.

Digital infrastructure, defined as the aggregate of digital technologies and the products and platforms they enable, is a crucial pillar and endogenous driver of rural digital development (68). In this study, its level is measured across three dimensions: network infrastructure, information service infrastructure, and digital financial infrastructure. The digitalization of the rural economy represents a key manifestation of digital rural development and an important basis for evaluating the effectiveness of rural digitalization. It encompasses four major dimensions: digital production, digital finance, digital marketing, and digital supply chains (50, 69, 70), all of which contribute to the transformation, upgrading, and sustainable development of the rural economy. Meanwhile, the digitalization of rural life refers to the application of digital technologies in transforming rural lifestyles, and this study uses the level of online cultural development, farmers’ digital consumption, and digital education as key indicators. Rural governance digitalization constitutes an important component of social governance, and improving governance capacity is essential for enhancing social governance efficiency. Through digital technologies, smart emergency management systems establish efficient response mechanisms, thereby providing strong support for rural revitalization. In addition, “Internet Plus” government services optimize rural governance by improving transparency and responsiveness. A digital rural ecosystem is a prerequisite for advancing rural digitalization, while the promotion of green production standards is an intrinsic requirement for building smart and environmentally sustainable rural communities. Drawing on relevant studies, this research incorporates indicators related to green living environments into the measurement system for rural ecological digitalization (71) to comprehensively reflect its level of development and outcomes.

#### 4.3.3 Mechanism variable

This study examines how rural digitalization influences the wellbeing of older adults in rural areas through three pathways: self-efficacy, social networks, and social trust. The proxy indicators and measurement methods for each mediating variable are as follows.

##### 4.3.3.1 Self-efficacy

This refers to an individual’s confidence in their ability to successfully complete specific tasks or achieve goals. It is measured using the CFPS questionnaire item, “How confident are you about the future?”, as a proxy variable (72). This indicator primarily reflects an individual’s positive expectations regarding their future circumstances and ability to act. Theoretically, it is linked to self-efficacy, which emphasizes an individual’s positive perception of their abilities and capacity to cope with future situations.

Higher Studies on older adults have found that higher levels of self-efficacy are significantly associated with optimism and subjective wellbeing (73), indicating that an individual’s positive assessment of their abilities is closely linked to a positive psychological outlook toward the future. Furthermore, related studies have found that self-efficacy is strongly associated with an individual’s future expectations (74), indicating that the belief in one’s abilities inherent in self-efficacy is intrinsically linked to perceptions and expectations regarding the future. Confidence in the future similarly reflects an individual’s positive expectations regarding future development and possibilities, exhibiting distinct future-oriented characteristics (75).

Although confidence in the future and self-efficacy are not entirely equivalent in conceptual meaning, the positive future expectations reflected by confidence in the future and the belief in one’s future capabilities inherent in self-efficacy share a theoretical connection. For individuals, maintaining confidence in the future not only reflects positive expectations regarding future possibilities but is also closely related to their ability to cope with future situations and potential challenges. This study treats confidence in the future as a proxy measure of self-efficacy rather than as a complete measure of this latent construct. Conceptual validity is further assessed by examining the systematic relationship between the proxy indicator and theoretically related variables. The relevant results are presented in Appendix Table A1.

##### 4.3.3.2 Social networks

Social networks refer to the social ties formed by individuals through ongoing interactions with relatives, friends, and neighbors. These ties are primarily reflected in the scope of social connections, frequency of interaction, and strength of relationships, which provide individuals with relational resources such as information exchange, mutual assistance, and social support (76).

In rural Chinese society, kinship, geographical ties, and acquaintance networks form the foundation of social interaction, while social exchanges and gift-giving serve as important means of maintaining social relationships (77, 78). Therefore, household gift-giving expenditures have social implications that extend beyond material consumption. The level of such expenditures reflects the extent to which households participate in social interactions and maintain relational ties and is closely associated with the strength of social relationships and social capital accumulation (77). Li and Ma (79) also support this relational nature, who regard household gift expenditures as an important economic manifestation of social interaction. More directly, expenditures on social obligations have been used to measure the social networks of rural residents (80), while the significant association between household gift-giving and social trust further demonstrates its relational nature (81).

Given that household expenditures on social gifts and gestures reflect both the extent of a household’s participation in social interactions and the effort invested in maintaining social relationships, this study constructs a regression analysis using the logarithm of household expenditures on social gifts and gestures as a proxy for the level of social networks among rural older residents (81). Based on the systematic relationship between the proxy indicator and theoretically relevant variables, supplementary tests of construct validity were conducted. The relevant results are presented in Appendix Table A2.

##### 4.3.3.3 Social trust

Social trust refers to the basic tendency to trust others and public entities that individuals develop through social interaction. This tendency varies across relational contexts, and different objects of trust do not necessarily carry entirely homogeneous meanings (82).

Since social trust is not directed at a single object but exists within various social relationships and interaction contexts, existing studies typically measure it across multiple trust objects, including parents, neighbors, strangers, government officials, and doctors, using a 0–10 rating scale to assess an individual’s level of trust (83, 84). Although different trust objects correspond to different social relationship contexts, they all reflect an individual’s propensity to trust specific social entities. Therefore, measuring trust across multiple social relationship dimensions can provide a relatively comprehensive portrayal of an individual’s overall level of social trust (85).

Based on this, the mean value of six CFPS indicators, namely, “trust in parents”, “trust in neighbors”, “trust in Americans”, “trust in strangers”, “trust in local government officials”, and “trust in doctors”-is used to measure the level of social trust (39), with scores ranging from 0 to 10. Reliability tests and exploratory factor analysis were conducted on the relevant items, and the results are presented in Appendix Tables A3, A4.

#### 4.3.4 Control variables

To improve the accuracy of the regression estimates and maintain consistency with existing research (38, 86), this study incorporates control variables at both the individual and household levels. At the individual level, the control variables include gender, age, educational attainment, political affiliation, religious beliefs, and marital status. At the household level, the variables include the logarithm of household income and size.

## 5 Empirical results

### 5.1 Baseline regression results

Based on the theoretical analysis and research design outlined above, this study employs a dual machine learning model to estimate the impact of rural digitalization on the wellbeing of older people in rural areas. The sample was divided using a 1:4 split ratio, and the Lasso CV algorithm was used to conduct predictive analysis for both the main and auxiliary regression models. The regression results are presented in Table 3.

**Table 3:** Baseline regression results on the impact of rural digitalization on the wellbeing of older adults in rural areas.

Variable name	(1)	(2)	(3)	(4)	(5)	(6)
	WB	WB	OJ	OC	OL	OE
DG	0.0434*** (0.010)	0.0427*** (0.010)	0.3215*** (0.042)	−0.1778*** (0.017)	0.0781*** (0.006)	0.0664*** (0.020)
Control variable term	YES	YES	YES	YES	YES	YES
The quadratic term of the control variable	NO	YES	YES	YES	YES	YES
Fixed effects for the year	YES	YES	YES	YES	YES	YES
Provincial fixed effects	YES	YES	YES	YES	YES	YES
Observed value	7,760	7,760	7,760	7,760	7,760	7,760

**p < 0.05, ***p < 0.01; values in parentheses are robust standard errors.

After controlling for the quadratic term, year fixed effects, and province fixed effects, Model (1) shows that the regression coefficient of rural digital development on the wellbeing of older adults is 0.0434, which is statistically significant at the 1% level. Building on Model (1), Model (2) further includes the quadratic terms of the control variables. The regression coefficient is 0.0427, which remains significantly positive and is very close to that of Model (1), indicating that the estimates of the core explanatory variables remain relatively stable even after accounting for potential nonlinear relationships among the control variables. Overall, the baseline DML results suggest a stable positive relationship between rural digital development and the wellbeing of rural older residents. This outcome can be attributed to two factors. First, rural digitalization has driven a process of “household digitalization,” enabling kinship ties that were previously constrained by urban–rural divides in digital space. Digital technology has become an important medium for maintaining intergenerational relationships among older adults. Children often support their parents in adapting to digital tools, and as older adults use these technologies to interact with their children, they not only benefit from improved communication but also gain emotional support and psychological fulfillment (87). Second, the rural digitalization of rural areas helps expand access to information, increases social participation among older adults in rural communities, and even enables them to obtain new forms of social recognition, which is of great significance for reshaping their social roles. Amid ongoing social change, rural older adults are increasingly redefining their identities through a process of identity reconstruction, shifting from passive recipients of care to active participants in society (88).

To further examine the impact of digitalization on different dimensions of wellbeing among older adults in rural areas, Models (3)—(6) estimate the regression coefficients for four sub-dimensions: employment of older adults, participation, living conditions, and the living environment. The results show that rural digitalization has a statistically significant positive effect on employment of older adults, quality of life, and living environment at the 1% level. Moreover, the magnitude of these positive effects on the sub-dimensions is greater than the overall effect on wellbeing. In contrast, the coefficient for older adults’ participation is statistically significant and negative at the 1% level. This result can be explained as follows: On the one hand, the development of digital villages is a key pathway for promoting inclusive rural development. Through the dissemination of agricultural technologies and educational programs, digital technologies enhance the skills and employment opportunities of vulnerable groups, thereby improving their social status and economic participation (89). Meanwhile, digital advancements facilitate the targeted flow of urban resources to rural areas by establishing mechanisms for collaboration among diverse stakeholders, significantly improving farmers’ access to resources and narrowing the digital divide between urban and rural areas. However, this process does not necessarily yield balanced and inclusive outcomes; older adults may be marginalized due to insufficient digital skills, and structural inequalities within rural society require long-term institutional intervention to be effectively addressed (68). Therefore, although the development of digital villages contributes to improvements in the wellbeing of rural older adults, its effect on enhancing their participation remains limited.

Given that the CFPS data include repeated observations of the same respondents across different years, we further examine the impact of fixed-effects specifications and clustering methods on the baseline results, with the relevant findings presented in Table 4. First, when standard errors are clustered at the provincial level, the estimated coefficients of the core explanatory variables remain positive, in addition to the existing provincial and annual fixed effects, although their statistical significance declines. This reduction in precision is expected when common shocks and error correlation within provinces are considered. Because the core explanatory variables are constructed at the province-year level and the sample comprises only 24 provincial clusters, an increase in standard errors is anticipated following provincial clustering.

**Table 4:** DML estimation results with fixed effects and cluster-robust standard errors.

Variables	(1) Baseline DML	(2) Province FE + Year FE	(3) Individual FE + Year FE	(4) Individual FE + Year FE
Standard errors	Robust	Province-clustered	Province-clustered	Individual-clustered
DG	0.0427*** (0.0100)	0.0281 (0.0477)	0.1009*** (0.0226)	0.1026***(0.0053)
Province FE	Yes	Yes	No	No
Individual FE	No	No	Yes	Yes
Year FE	Yes	Yes	Yes	Yes
Observations	7,760	7,760	7,760	7,760
Individuals	4,241	4,241	4,241	4,241
Provinces	24	24	24	24

Values in parentheses denote standard errors. Model (1) employs heteroscedasticity-robust standard errors, whereas Models (2) and (3) utilize province-cluster-robust standard errors, and Model (4) applies individual-cluster-robust standard errors. All models incorporate fixed-year effects. The individual fixed-effects model accounts for time-invariant individual heterogeneity through within-individual mean adjustment. ***p < 0.01.

Furthermore, to account for time-invariant individual heterogeneity among the same respondents, we employ individual and year fixed effects for longitudinal estimation. The estimated coefficient for digital development is 0.1009 with provincial cluster-robust standard errors, which is significantly positive at the 1% level. This result indicates that after controlling for unobservable, time-invariant individual heterogeneity, the positive relationship between rural digital development and the wellbeing of rural older residents persists.

In addition, we employ individual cluster-robust standard errors to account for correlation in disturbance terms across different years for the same respondent. The results show that the estimated coefficient for the core explanatory variable is 0.1026, which is very close to the 0.1009 obtained under provincial clustering, with the statistical significance remaining unchanged. Thus, the point estimate is insensitive to the clustering level; changing the clustering level primarily affects the standard error but does not alter the positive estimated relationship between digital development and the wellbeing of rural older residents.

Therefore, we retain the DML model with provincial and year fixed effects as the baseline and further examine the impact of panel dependency structures on statistical inference through supplementary analyses, including provincial clustering, individual fixed effects, and individual clustering. Notably, we do not select estimation results based on statistical significance; rather, we report results according to the identification sources and error dependency structures of each model. The analysis indicates that the core positive relationship remains robust after controlling for individual fixed-effects heterogeneity.

### 5.2 Robustness tests

To further verify the robustness of the baseline regression results, this study conducts robustness tests from three perspectives: excluding outliers, adjusting the sample split ratio, and replacing the core estimation algorithm.

First, outliers in the regression sample, particularly for variables constructed using composite weighting methods, may lead to biased estimates. Therefore, in the baseline regression, all variables except the core explanatory variable were winsorized at the 1st and 99th percentiles; values above the upper bound and below the lower bound were replaced, and the adjusted dataset was used for the regression analysis. As shown in columns (1) and (2) of Table 5, after addressing outliers, although the magnitude of the regression coefficient for the impact of rural digital development on the wellbeing of older adults changed, it remained significantly positive at the 1% level. This confirms the robustness of the baseline regression results.

**Table 5:** Robustness tests.

Variable name	(1)	(2)	(3)	(4)	(5)	(6)
	1% tail	5% discount	Kfolds = 3	Kfolds = 8	Svm	Gradboost
DG	0.0257*** (0.008)	0.0434*** (0.010)	0.0432*** (0.010)	0.0423*** (0.010)	0.1241*** (0.003)	0.0232*** (0.007)
Control variable term	YES	YES	YES	YES	YES	YES
The quadratic term of the control variable	YES	YES	NO	YES	YES	YES
Fixed effects for the year	YES	YES	YES	YES	YES	YES
Provincial fixed effects	YES	YES	YES	YES	YES	YES
Observed value	7,760	7,760	7,760	7,760	7,760	7,760

***p < 0.01.

Second, to avoid discrepancies in the results caused by the sample split ratio, this study adjusts the split ratio of the double machine learning model from the baseline of 1:4 to 1:2 and 1:7 to examine the potential impact of this parameter on the findings. As shown in columns (3) and (4) of Table 4, after adjusting the sample split ratio, the significance of the effect of rural digital development on the wellbeing of older adults remains significant. The estimated coefficients remain positive and are similar in magnitude to those in the baseline regression, thereby confirming the robustness of the original conclusions.

Third, to ensure that the choice of algorithm does not affect the reliability of the estimates, the original Lasso regression is replaced with support vector machines and gradient boosting methods to further examine the robustness of the baseline results. As shown in columns (5) and (6) of Table 4, the impact of rural digitalization remains significantly positive at the 1% level. This indicates that digitalization continues to exert a significant positive effect on the wellbeing of older adults in rural areas, thereby confirming the validity of the baseline regression results.

### 5.3 Endogeneity tests

The above analysis provides preliminary evidence of the association between rural digital development and the wellbeing of older adults in rural areas. However, this relationship is inherently complex and may be affected by reverse causality, omitted variable bias, and endogeneity. To address these concerns, this study employs a partial linear instrumental variable model within the double machine learning framework. The instrumental variables consist of the logarithm of rural landline telephone subscriptions in 2008 (90) and its interaction with the level of rural digital development.

The instrumental variables selected in this study have a sound theoretical and empirical basis in terms of relevance and exogeneity, as detailed below.

#### 5.3.1 Instrumental variable

The interaction term between the logarithm of the number of rural landline phones in 2008 and the ex ante variable for rural digital infrastructure development (t + 1) is used as an instrumental variable. Regarding relevance, landline networks constitute a key component of early information and communications infrastructure. Historical communications infrastructure influences subsequent Internet access and digital economy development through network facilities, technological foundations, and user habits. Previous studies used historical landline penetration rates to identify regional digital economic development and established their relevance through first-stage or weak- instrument diagnostics (91–93). Furthermore, because historical landline indicators primarily capture cross-sectional differences, interacting these indicators with digital development variables that vary over time allows the instrumental variable to capture both historical differences in regional communication endowments and temporal changes in digital development. This identification strategy, which constructs instrumental variables using historical exposure levels and time-varying factors, has established precedents in empirical research (94) and has also been adopted in recent digital economy studies (91, 93). Regarding exogeneity, the number of rural landline telephones in 2008 was determined prior to the 2016–2022 study period and is therefore unlikely to be affected by changes in the wellbeing of rural older residents during the study period. Additionally, this study controls for individual- and household-level characteristics, provincial fixed effects, and year fixed effects to account for persistent regional characteristics and common time shocks, thereby reducing the likelihood that historical communication infrastructure affects current wellbeing through alternative channels. The variable representing rural digital infrastructure development one period prior in the instrumental variables model is denoted as t + 1. This study does not claim that this variable is exogenous solely because of its temporal positioning; rather, it treats it as an interactive component that captures dynamic digitalization changes. This interaction term provides a theoretically grounded instrument with respect to both relevance and exogeneity.

The regression results in Table 6 indicate that the estimated coefficient for rural digital development is 0.2468, after incorporating instrumental variables based on historical telecommunications infrastructure and rural digital development trends, which is significantly positive at the 1% level and consistent within the baseline regression. These findings indicate that the positive association between rural digitalization development and the wellbeing of rural older residents remains robust after accounting for potential endogeneity arising from omitted variables and bidirectional causality, thereby providing further support for the baseline results.

**Table 6:** Endogeneity tests.

Variable name	(1)
	IV: Construct an interaction term using the logarithm of rural landline telephone numbers from 2008 and the level of rural digital development from the previous period
DG	0.2468*** (0.033)
Control variable term	YES
Quadratic term of the control variable	YES
Fixed effects for the year	YES
Provincial fixed effects	YES
Observed value	7,760

***p < 0.01.

### 5.4 Analysis of mechanisms of action

The preceding findings indicate that rural digital development is significantly associated with improved wellbeing among rural older residents. To further examine the underlying mechanisms, this study adopts the approach proposed by Jiang (95). Building on existing theoretical frameworks, this study focuses on three potential channel variables, namely, self-efficacy, social networks, and social trust, and examines how rural digital development is associated with these variables and, in turn, with the wellbeing of rural older residents. Given that mediating variables may be endogenous in observational data, mechanism analyses should be grounded in clear theoretical foundations and should carefully consider the conditions required for causal identification (95–97). Accordingly, this study examines the potential mechanisms linking rural digital development to the wellbeing of older residents in rural areas through self-efficacy, social networks, and social trust.

Column (1) of Table 7 shows that rural digitalization has a significant positive effect on the self-efficacy of older adults in rural areas, these results provide empirical support for the self-efficacy mechanism proposed in Hypothesis H3. Specifically, a one-unit increase in rural digitalization is associated with a 0.1260-unit increase in self-efficacy. Guided by the theory of active aging, rural digital development facilitates the integration of older adults into the digital society. By empowering them and strengthening their sense of agency, self-efficacy in adapting to and participating in digital environments is enhanced (98). Furthermore, self-efficacy is an important psychological resource that enables older adults to cope with environmental changes and maintain a sense of agency, thereby influencing their social connections, psychological wellbeing, and health status. Higher levels of self-efficacy can reduce social isolation and loneliness while enhancing the ability of older adults to establish and maintain social connections (45). Moreover, self-efficacy, together with social support, can strengthen a sense of hope and promote positive psychological states (44) and is closely associated with wellbeing-related outcomes such as community participation and self-rated health (49). Therefore, higher self-efficacy may strengthen rural older residents’ sense of control, social participation, and ability to adapt to their environment, thereby contributing to improved wellbeing.

**Table 7:** Results of the analysis of action channels.

Variable name	(1)	(2)	(3)
	Self-efficacy	Social media	Level of social trust
DG	0.1260*** (0.013)	0.4626*** (0.037)	0.5675*** (0.020)
Control variable term	YES	YES	YES
The quadratic term of the control variable	YES	YES	YES
Fixed effects for the year	YES	YES	YES
Provincial fixed effects	YES	YES	YES
Observed value	7,760	7,760	7,760

***p < 0.01.

Column (2) of Table 7 shows that rural digital development significantly enhances the social network levels of rural older residents, providing empirical support for Hypothesis H4. Specifically, a one-unit increase in rural digitalization leads to a 0.4626-unit increase in the social networks of older adults in rural communities. Social networks, which are grounded in interaction and communication, enable individuals to gain subjective experiences such as belonging, trust, and mutual support, thereby enhancing overall wellbeing. Emerging internet-based industries and business models continue to strengthen connectivity in rural areas, reflecting the steady progress of rural digitalization development. As social networks expand in depth and their breadth and communication channels improve, they provide older adults with richer opportunities for interaction and greater relational resilience (55). Furthermore, stable and effective social networks provide older adults with ongoing emotional support, access to information and resources, and assistance with daily activities. Relatively extensive family and friends can enhance cognitive function and quality of life among older adults (52). The size of social networks, frequency of social contact, and quality of social support are also closely associated with the risk of depression among older adults, while stable social connections can help alleviate social isolation and negative psychological states (57). Strong social support can promote social integration and improve the quality of life among older adults (56). Therefore, rural digital development may enhance access to emotional support and resources among rural older residents by expanding their social networks, alleviating loneliness and social isolation, and contributing to improved wellbeing.

Column (3) of the Table 7 shows that the development of rural digitalization has a significant positive impact on the level of social trust among older adults in rural areas; these results provide empirical support for the social trust pathway proposed in Hypothesis H2. Specifically, a one-unit increase in rural digitalization is associated with a 0.5675-unit increase in social trust, an effect that is statistically significant at the 1% level. In the digital age, digital platforms have become important tools for rural governance. The development of digital villages strengthens connections among stakeholders and enhances oversight mechanisms by improving information transparency and promoting greater interaction and public participation. This, in turn, can improve rural governance effectiveness and foster a social environment characterized by greater mutual trust (40). Furthermore, social trust reduces uncertainty in social interactions, enhances the sense of security and social belonging, and increases their willingness to participate in social activities, thereby providing an important foundation for maintaining social cohesion and integration. Higher levels of trust in social groups, markets, local governments, and general social trust are significantly and positively associated with life satisfaction among rural older adults, indicating that greater social trust contributes to more positive life evaluations (99). Social trust is also closely linked to the health status of older adults and serves as an important psychosocial link between subjective social status and health (38). Moreover, social trust is a key social factor influencing life satisfaction among middle-aged and older adults in China and is closely associated with wellbeing (100). Therefore, rural digital development may enhance rural older residents’ sense of social security and integration by strengthening social trust, thereby contributing to improved wellbeing.

### 5.5 Heterogeneity analysis

The preceding analysis indicates that rural digitalization development in rural areas significantly improves the wellbeing of rural older residents; however, the average effect estimated for the overall sample may mask differences across subgroups. Therefore, this study conducts a heterogeneity analysis based on educational attainment, intergenerational living arrangements, and regional distribution.

Educational attainment was divided into two groups: individuals with at least a high school education (Edu = 1) and those with less than a high school education (Edu = 0). As shown in columns (1) and (2) of Table 8, the positive association between rural digital development and the wellbeing of rural older residents is more pronounced among those with lower education levels, whereas it is insignificant among those with higher levels of education. This discrepancy may arise because groups with lower levels of education have relatively limited channels for accessing information and utilizing digital resources. Consequently, they may derive greater marginal benefits from improvements in regional digital infrastructure and digital public services. In contrast, individuals with higher levels of education generally possess stronger capabilities to access information and utilize digital resources; thus, the marginal benefits they derive from improvements in regional digital development may be relatively limited (101).

**Table 8:** Results of heterogeneity analysis.

Variable name	(1)	(2)	(3)	(4)	(5)	(6)	(7)
	High level of education	Low level of education	Living with one’s children	Does not live with their children	East	Central	The west
DG	0.0545 (0.034)	0.0409*** (0.010)	0.0465*** (0.013)	0.0414** (0.017)	0.1291*** (0.004)	0.4603*** (0.008)	0.4828*** (0.015)
Control variable term	YES	YES	YES	YES	YES	YES	YES
The quadratic term of the control variable	YES	YES	YES	YES	YES	YES	YES
Fixed effects for the year	YES	YES	YES	YES	YES	YES	YES
Provincial fixed effects	YES	YES	YES	YES	YES	YES	YES
Observed value	700	7,060	5,402	2,358	3,370	2,422	1968

**p < 0.05; ***p < 0.01.

Living with at least one child is defined as ‘living with children’, whereas the alternative is defined as “not living with children”. The regression results for these two living arrangements are presented in columns (3) and (4) of Table 8. As shown, compared with older adults living apart from their children, rural digital development has a more significant positive effect on the wellbeing of those living with their children. One possible explanation is that co-residence with children provides older adults with greater intergenerational support in using the local digital environment and digital services. Existing research indicates that children can help older adults adapt to and use digital technologies through day-to-day guidance (22), making it easier for older adults to access support. Such support includes financial assistance, daily care, and emotional comfort, thereby enhancing their wellbeing. However, improvements in transportation and Internet technologies also enable older adults living apart from their children to receive substantial support through social media platforms and other digital channels (87). This study does not directly measure the individual digital skills or literacy of older adults; therefore, the explanations above are provided solely to illustrate possible sources of heterogeneity.

The study area is divided into three major regions: East, Central, and West. Based on the results in columns (5) and (6) of Table 8, notable regional differences exist in the association between rural digital development and the wellbeing of older adults. Although rural digital development is positively associated with wellbeing across all three regions, it is more pronounced in the central and western regions. This may be because rural areas in the central and western regions generally have lower levels of digital infrastructure than those in the eastern region. Consequently, improvements in the digital environment may generate greater marginal benefits in relatively less-developed areas. In contrast, the eastern regions already have more advanced digital environments, leaving relatively less room for additional digital dividends (102).

## 6 Discussion

This study identifies a significant positive association between rural digitalization and the wellbeing of rural older adults. It further demonstrates that digitalization is not merely a technical tool but is increasingly becoming an important source of social empowerment, closely associated with rural older adults’ social participation, access to resources, and opportunities for self- realization in rural communities. Consequently, the significance of rural digital development extends beyond traditional infrastructure improvement, as it profoundly reshapes how older adults in rural areas engage with society and the underlying mechanisms that support their wellbeing.

First, regarding the overall positive association between rural digital construction and the wellbeing of older adults in rural areas, this study not only confirms previous findings that digitalization improves subjective wellbeing (23) but also extends the theoretical scope of these effects. Unlike prior studies that largely focus on unidimensional indicators such as subjective wellbeing, mental health, or quality of life (103, 104), this study, grounded in the WHO framework for Active Aging and informed by the AAI’s multidimensional measurement approach, this study develops a comprehensive wellbeing evaluation system tailored to the context of rural China, encompassing four dimensions: employment, participation, quality of life, and living environment. The findings show that rural digitalization development significantly improves the overall wellbeing of older adults in rural areas. This effect is reflected not only in improved convenience and access to public services but also in enhanced digital social integration, social connectedness, and access to developmental opportunities. This indicates that efforts to enhance the wellbeing of older adults in rural areas are shifting from traditional “safety-net” welfare toward a greater emphasis on social integration, personal development, and quality of life in the context of digitalization. However, further analysis shows that the current rural digitalization has a relatively limited effect on promoting the participation of older adults, indicating that digital development has so far focused mainly on “empowering daily life services”, with room for improvement in stimulating deeper forms of social engagement. Therefore, future policies should not only continue to strengthen rural digital infrastructure and inclusive digital services but also prioritize the creation of older-friendly environment for social participation. This includes expanding opportunities for older adults in rural areas to engage in community governance, social interaction, and public affairs, thereby advancing rural digitalization from “protective empowerment” to “participatory empowerment” and systematically enhancing their overall wellbeing.

Second, the analysis of the underlying mechanisms indicates that rural digital development significantly enhances the self-efficacy of rural older residents, expands their social networks, and strengthens social trust. Higher self-efficacy can enhance the sense of control of older adults, promote positive psychological states, and improve their ability to adapt to environmental changes (44, 45). Stable social networks provide ongoing emotional support and access to information and resources, thereby alleviating social isolation and improving quality of life (52, 57). Furthermore, higher levels of social trust can enhance feelings of security, life satisfaction, and social integration (38, 99, 100). Thus, the benefits of rural digital development extend beyond improved access to technology and services; they also operate through enhanced agency, stronger social relationships, and greater interpersonal trust among older adults. Future efforts should strengthen the inclusiveness of the digital environment and expand community-level support systems to better harness the potential of digital development to promote active aging.

Finally, the heterogeneity analysis further reveals that the association between rural digitalization and wellbeing varies across different groups. The positive association is more pronounced among rural older adults with lower educational attainment, those living with their children, and those residing in the central and western regions. These findings indicate that digitalization may generate greater benefits for groups with relatively limited access to traditional resources. Specifically, digitalization can enhance the learning ability and receptiveness of individuals with low educational attainment (51). Furthermore, the stronger positive association observed among older adults living with their children highlights the important role of intergenerational family support in the process of digital inclusion (105). This indicates that digital reverse mentoring is not merely a process of acquiring technical skills but also a mechanism for intergenerational emotional interaction and social support. This finding also indicates that the development of digital literacy among older adults in rural areas is not solely dependent on individual learning but is deeply embedded in family structures, social relationships, and regional development conditions. In future policy design aimed at improving the wellbeing of older adults in rural areas, the distinct characteristics of different groups must be fully considered, and younger family members must be encouraged to provide daily guidance and emotional support through “digital mentoring,” thereby helping older adults adapt to digital technologies. More importantly, older adults should be encouraged to take the initiative in learning, actively engage with digital tools, and be supported in continuously improving their digital literacy.

## 7 Conclusions and implications

### 7.1 Research findings

Amid the twin trends of global population aging and rural digitalization, building a more inclusive digital society and ensuring that older adults in rural areas share in development outcomes have become common challenges worldwide. This study uses micro-level matched data from the CFPS and the Provincial Digital Development Index for 2016, 2018, 2020, and 2022. This study uses a double machine learning model to examine the impact of rural digitalization on the wellbeing of older adults in rural areas and the mechanisms of Action and Heterogeneity. First, the findings show that rural digitalization is significantly positively associated with the overall wellbeing of rural older residents. This indicates that a favorable environment for regional digital development may create favorable conditions for rural older adults to access public services, participate in social activities, and improve their living conditions, effectively promotes older adults’ health, social participation, and overall wellbeing, highlighting the strong potential of digital technology in empowering rural populations. Second, an analysis of the underlying mechanisms indicates that rural digitalization significantly enhances the self-efficacy of rural older residents, expands their social networks, and strengthens social trust. Based on the active aging framework and existing empirical evidence, these factors may serve as important channels through which rural digitalization contributes to improved wellbeing among rural older residents. Third, the wellbeing association of rural digitalization varies significantly across different group characteristics and regional endowments. The effects are more pronounced among older adults with lower education levels, those with moderate or lower incomes, those living with their children, and those residing in central and western regions. Consistent with digital divide and resource inequality theories, rural digitalization appears to provide greater benefits for groups with relatively limited access to information and social resources. This finding underscores the important role of rural digitalization in promoting social equity and empowering vulnerable groups.

### 7.2 Limitations of the study and future research directions

Although this study provides a relatively systematic analysis of the relationship between rural digitalization and the wellbeing of older adults in rural areas, it has certain limitations that should be addressed and further refined in future research.

First, this study excluded regions with incomplete data during the empirical analysis stage due to limitations in data availability for rural areas in some Chinese provinces; consequently, the final sample does not cover all rural areas. Future research could improve the spatial generalizability and policy relevance of the findings by integrating data from multiple sources and conducting targeted surveys in underdeveloped rural regions.

Second, while this study mainly examines the impact of rural digitalization on the wellbeing of older adults from the perspective of active aging, further exploration of deeper social relationship factors, such as intergenerational family interactions and the positive effects of digital technologies on older adults, is warranted. Future research could address this dimension by developing a comprehensive and systematic analytical framework and deepening the understanding of how intergenerational support and rural digital initiatives jointly enhance the wellbeing of older adults.

## Data Availability

The raw data presented in the study is publicly available at Zenodo at: https://doi.org/10.5281/zenodo.20538765.
