# Supplementary material for: Rural digitalization and the multidimensional wellbeing of older adults in China: evidence from the active aging framework
Source: Front Public Health. 2026 Oct 19;14:1900178. doi: 10.3389/fpubh.2026.1900178 (PMC13728775; doi:10.3389/fpubh.2026.1900178)
Supplement: Supplementary file 1 [file Supplementary_file_1.pdf]

Supplementary Material

Appendix A:

Self-efficacy and social network adoption are measured using single-item proxy indicators in this study; therefore, the traditional Cronbach's alpha, which is based on internal consistency across multiple items, is not applicable to these measures. Therefore, this study employs tests of associative validity using theoretically related variables as supplementary evidence of construct validity.

Table A1: Supplementary construct validity tests for the self-efficacy proxy variable

Validation Type	Proxy Measure	Theoretical Concept	Validation Measure	Theoretical Expectation	Test Results
Theoretical Construct Validity Test	Level of Confidence in One's Future	Capacity for Subjective Conduct	Efficiency in Work	Positive Correlation	ρ = 0.1714***
Adjusted Association Test	Level of Confidence in One's Future	Capacity for Subjective Conduct	Efficiency in Work	Positive Correlation	β = 0.2733***

***P < 0.01.

Table A1 reveals a significant positive correlation between "level of confidence in one's future" and "efficiency in performing tasks," a subjective indicator of behavioral competence aligned with the theoretical framework. After controlling for covariates, this association remains significant, thereby providing supplementary evidence of construct validity for using "level of confidence in one's future" as a proxy for self-efficacy.

Table A2: Construct validity test for social network proxy variables

Panel A: Spearman's Correlation Test

Proxy Indicators	Child-Parent Relationships	Child-Parent Connections	Child-Parent Networks
ln_gift	0.0633***	0.1428***	0.1256***
N	3,737	3,737	3,737

Panel B: Ordered Logit Test with Covariate Controls

Variables	Child-Parent Relationships	Child-Parent Connections	Child-Parent Networks
ln_gift	0.0442***	0.0477***	0.0585***
Robust SE	(0.0113)	(0.0116)	(0.0111)
Control Variables	Yes	Yes	Yes
Age	Yes	Yes	Yes

Variables	Child-Parent Relationships	Child-Parent Connections	Child-Parent Networks
Gender	Yes	Yes	Yes
Education	Yes	Yes	Yes
Marital status	Yes	Yes	Yes
Health	Yes	Yes	Yes
Observations	3,483	3,479	3,479

***P < 0.01.

Table A2 demonstrates that, after controlling for various individual characteristics, household expenditure on social gifts and gestures maintains a stable and significant positive association with indicators reflecting social relationships and social ties. Consequently, these findings further support the construct validity of using such expenditure as a proxy for social networks.

To evaluate the measurement of the social trust index, this study performed reliability tests and exploratory factor analysis on the six constituent trust items. Specifically, Cronbach's alpha was used to assess the internal consistency of the items, whereas the KMO test and Bartlett's test of sphericity were used to determine the suitability of the data for factor analysis. Subsequently, principal component analysis was employed to extract factors, and Promax oblique rotation was used to examine the latent structure of the items. The detailed results are presented in Tables A3 and A4.

Table A3: Reliability and factor analysis fit tests for social trust levels

Indicators	Value
Cronbach's α	0.677
KMO Statistic	0.704
Bartlett's Test of Sphericity χ^2	7,983.00
Degrees of freedom（df）	15
P-value	<0.001

The null hypothesis for Bartlett's test of sphericity posits that the correlation matrix is an identity matrix.

As shown in Table A3, Cronbach's alpha for the six social trust items was 0.677, indicating acceptable reliability. The KMO statistic was 0.704, indicating adequate commonality among the items. Furthermore, Bartlett's test of sphericity was significant (chi-squared= 7,983.00, df = 15, P < 0.001), thereby rejecting the null hypothesis that the correlation matrix is an identity matrix. These results confirm significant inter-item correlations among the items, demonstrating that the data are suitable for further factor analysis.

Table A4: Exploratory factor analysis of social trust levels

Item	In-group social trust (Factor 1)	Out-group social trust (Factor 2)	Uniqueness
Trust in Parents	0.657	-0.167	0.591
Trust in Neighbors	0.739	0.036	0.44
Trust in Officials	0.719	0.134	0.421
Trust in Doctors	0.780	0.006	0.389
Trust in Strangers	0.008	0.837	0.296
Trust in Americans	0.033	0.825	0.306
Characteristic Root	2.344	1.213	—
Percentage of Variance Explained	39.06%	20.22%	—
Cumulative Percentage of Variance Explained	59.28%	—	—

Factors were extracted using principal component analysis with Promax oblique rotation. The factor loadings presented in the table are post-rotation values, whereas the eigenvalues and percentages of variance explained correspond to the initial factor extraction stage.

Table A4 presents the results of the exploratory factor analysis for the six social trust measuring items. Factor extraction was conducted using principal component analysis with Promax oblique rotation to accommodate potential correlations among the different dimensions of trust. Based on the Kaiser criterion, factors with eigenvalues greater than 1 were retained, yielding two factors. Factor 1 had an eigenvalue of 2.344 and explained 39.06% of the variance, whereas Factor 2 had an eigenvalue of 1.213 and explained 20.22% of the variance. The cumulative variance explained by these two factors was 59.28%, indicating that they account for 59.28% of the total variance in the six items.

Based on the rotated factor loadings, trust in parents, neighbors, grassroots officials, and doctors primarily loaded onto Factor 1, with loadings of 0.657, 0.739, 0.719, and 0.780, respectively, characterizing this factor as in-group social trust. Conversely, trust in strangers and Americans loaded primarily onto Factor 2, with loadings of 0.837 and 0.825, respectively, characterizing it as out-group social trust. The rotated factor loadings indicate that each item primarily loads onto its corresponding factor, with generally low cross-loadings, demonstrating a clear factor affiliation. Overall, the six trust items exhibit a distinct latent structure. While the items share certain commonalities, differences exist across the relational domains of the trust objects, which aligns with the theoretical understanding that social trust possesses domain-specific attributes. Given that this study examines the mediating role of overall social trust, the baseline regression employs the arithmetic mean of the six trust items to construct the overall social trust.

Appendix B:

While retaining the innovative design of the original multidimensional well-being index, we conducted a more rigorous examination of potential conceptual overlap and constructed a more conservative "Core Older Adults' Well-being Index" for robustness testing, thereby enhancing the robustness and explanatory power of the findings. The specific details are as follows:

Table B1: Robustness Tests Based on the Core Well-being Index

**Variable name**	**(1)**<br>**WB**	**(2)**<br>**WB**	**(3)**<br>**OJ**	**(4)**<br>**OC**	**(5)**<br>**OL**
DG	0.0487***	0.0479***	0.3215***	-0.1778***	0.1014***
	(0.0076)	(0.0076)	(0.0415)	(0.0170)	(0.0061)
Control variable term	YES	YES	YES	YES	YES
The quadratic term of the control variable	NO	YES	YES	YES	YES
Fixed effects for the year	YES	YES	YES	YES	YES
Provincial fixed effects	YES	YES	YES	YES	YES
Observed value	7760	7760	7760	7760	7760

***P<0.01.

Table 3 presents the baseline regression results for the impact of rural digitalization on the well-being of older adults in rural areas. A comparison with the results in Table B1 is as follows:

First, robustness tests using the Core Well-being Index support the main conclusions of this study. After excluding three indicators, namely, internet usage, frequency of contact with family and friends, and satisfaction with the government, which may conceptually overlap with digital development, the eliminated effect of rural digital development on the Core Well-Being Index remained significantly positive ($\beta = 0.0487$, p $\beta = 0.0479$) in the model including a quadratic term. This finding indicates that the core results are not driven by mechanical correlations arising from the inclusion of digital-related indicators in the well-being index.

Second, the absolute coefficient values increased slightly relative to the baseline regression. The coefficient for DG in the core well-being index model increased from 0.0434 to 0.0487. This result further reinforces the reliability of the conclusions, confirming that the positive effect of rural digital development on the well-being of older adults is genuine rather than a statistical artifact caused by overlapping indicators.

Third, the results vary across dimensions. The coefficients for older adults' employment and participation remain identical to those in the baseline regression because these two dimensions do not require adjustment for environmental indicators.

The coefficient for older adults’ quality of life increased from 0.0781 to 0.1014. This increase may occur because, after excluding digitalization-related environmental indicators, the remaining indicators, such as physical health, mental health, cognitive function, and social security, more accurately reflect the indirect positive impact of digitalization on quality of life through non-digital channels.
